# PAX7 is required for patterning the esophageal musculature

**DOI:** 10.1186/s13395-015-0068-0

**Published:** 2015-12-03

**Authors:** Daisuke Chihara, Anthony I. Romer, C. Florian Bentzinger, Michael A. Rudnicki, Robert S. Krauss

**Affiliations:** Department of Developmental and Regenerative Biology, Mount Sinai School of Medicine, New York, NY 10029 USA; Graduate School of Biological Sciences, One Gustave L. Levy Place, Icahn School of Medicine at Mount Sinai, New York, NY 10029 USA; Regenerative Medicine Program, Ottawa Hospital Research Institute, 501 Smyth Road, Ottawa, K1H 8L6 ON Canada; Department of Cellular and Molecular Medicine, University of Ottawa, 451 Smyth Road, Ottawa, K1H 8M5 ON Canada; Present address: Department of Genetics and Development, Columbia University, 701 West 168th Street, HHSC 1602, New York, NY 10032 USA; Present address: Nestlé Institute of Health Sciences, EPFL Campus, 1015 Lausanne, Switzerland

**Keywords:** Esophageal myogenesis, Megaesophagus, Pax7, Skeletal muscle, Smooth muscle, Cell proliferation, Frontal expansion

## Abstract

**Background:**

The mammalian esophageal musculature is unique in that it makes a transition from smooth to skeletal muscle, with most of this process occurring after birth. In order to better understand the mechanisms that control esophageal musculature development, we investigated the roles in this process of the paired box transcription factor, PAX7, a principal regulator of skeletal myogenic progenitor cells. Previous studies showed that *Pax7* is important for determining the esophageal muscle composition.

**Results:**

We characterized the postnatal development of the esophageal musculature in *Pax7*^*−/−*^ mice by analyzing morphology, muscle composition, and the expression of markers of myogenesis, cell proliferation, and apoptosis. *Pax7*^*−/−*^ mice displayed megaesophagus with a severe defect in the postnatal developmental process whereby esophageal smooth muscle is replaced by skeletal muscle. *Pax7*^*−/−*^ esophagi have substantially reduced skeletal muscle, most likely due to diminished proliferation and premature differentiation of skeletal muscle precursor cells. This impaired the proximal-to-distal progression of skeletal myogenesis and indirectly affected the patterning of the smooth muscle-containing portion of the esophageal musculature.

**Conclusions:**

Postnatal patterning of the esophageal musculature appears to require robust, PAX7-dependent cell proliferation to drive the proximal-to-distal progression of skeletal myogenesis. This process in turn influences distal smooth muscle morphogenesis and development of the mature pattern of the esophageal musculature.

**Electronic supplementary material:**

The online version of this article (doi:10.1186/s13395-015-0068-0) contains supplementary material, which is available to authorized users.

## Background

The musculature of the esophagus controls passage of food into the stomach by waves of peristaltic contractions. The lower esophageal sphincter (LES) is a bundle of smooth muscles at the distal end of the esophagus, where it meets the stomach. During swallowing, tonic smooth muscles of the LES relax briefly in order to allow passage of food into the stomach [1]. Esophageal muscles are adversely affected in a number of human disorders, including myotonic dystrophy, oculopharyngeal muscular dystrophy, and the inflammatory myopathies; this may lead to dysphagia, regurgitation, choking while eating, and other symptoms. Disorders of LES function include gastroesophageal reflux disease (GERD) and achalasia, the latter characterized by impaired relaxation of the LES and perturbed peristalsis, often resulting in megaesophagus. The primary cause of achalasia is defective signaling between the NO-producing inhibitory myenteric neurons and neighboring smooth muscle cells (SMCs) of the LES [2, 3]. Although studies have identified several mutant mouse lines that display these esophageal defects, their etiologies are poorly understood.

The mammalian esophageal musculature is unique in that it makes a transition from smooth to skeletal muscle, with most of this process occurring after birth. The esophagus is ensheathed by the muscularis externa (ME) [1, 4, 5]. Skeletal muscle comprises the proximal portion of the ME and is critical for swallowing and proximal waves of peristalsis. Smooth muscle surrounds the distal portion, including the esophagogastric junction, which harbors the LES. During mammalian development, the ME initially comprises only smooth muscle. In the mouse, skeletal muscle precursors are first detected in the proximal ME at embryonic day (E) 13 [6]. Over the next 3 weeks of life, smooth muscle is replaced by skeletal muscle in a proximal-to-distal manner, and the adult ME pattern is nearly completed by postnatal day (P) 14 [7–9]. Human esophageal myogenesis is similar, although smooth muscle is maintained in a more proximal position than in the mouse, but is still restricted to the distal one-third of the esophagus [10]. Interestingly, the amount and distribution of skeletal muscle varies among individuals [11, 12]. The developmental and cell biological mechanisms that underlie the smooth to skeletal muscle replacement process have been controversial, with multiple mechanisms proposed [7–9, 13–15] (reviewed in ref. [13]). Rishniw et al. [9] were the first to suggest that some sort of distal compaction of smooth muscle cells was important in the replacement of smooth muscle with skeletal muscle. However, the mechanisms for this process remained unclear.

Our previous study provided insight into mechanisms that control morphogenesis of the esophageal ME [13]. We showed that mice lacking the multifunctional cell surface receptor Cdo (also called Cdon) have a defect in the postnatal developmental process whereby esophageal smooth muscle is replaced by skeletal muscle. Analyses of various skeletal myogenic markers revealed that proliferative skeletal muscle progenitor cells migrated in a transition zone (TZ) along the length of the esophagus in a proximal-to-distal manner, leaving differentiated myofibers in its wake. Distal to the TZ, smooth muscle fascicles underwent a morphogenetic process whereby they changed their orientation relative to each other and to the lumen. In a distal-to-proximal manner, smooth muscle fascicles altered their orientation from parallel to the lumen to nearly perpendicular to the lumen. Upon completion of the process, smooth muscle occupied only the most distal part of the esophagus and the LES. *Cdo*^*−/−*^ mice had no defect in skeletal myogenesis or in the numbers of various skeletal muscle progenitor cell types in the TZ. Rather, they were specifically defective in SMC fascicular reorientation, leading to ectopic, proximal fascicles displaying an inappropriate orientation and an aberrantly proximal skeletal-smooth muscle boundary [13]. Importantly, *Cdo*^*−/−*^ mice also displayed megaesophagus and achalasia, with a smooth muscle cell-intrinsic defect in NO-mediated LES relaxation. Taken together, our previous findings identified a mechanism of ME maturation and provided a link between esophageal morphogenesis and motility disorders. Although the ME maturation defect in *Cdo*^*−/−*^ mice is almost certainly smooth muscle cell-autonomous, a role for TZ-based skeletal myogenesis in promoting the fascicular reorientation process seems likely.

Multiple mechanisms have also been proposed for the developmental origin of esophageal skeletal muscles (ESMs). Minchin et al. reported that, similar to trunk and limb muscles, ESMs arise from Pax3/Pax7-expressing cells that originate in somites [16]. However, Gopalakrishnan et al. have recently demonstrated that ESMs are derived from the cardiopharyngeal mesoderm, and their development is dependent on expression of *Tbx1* and *Isl1*, genes important for specific muscles of the head but not the trunk [17]. These factors lie upstream of the myogenic bHLH factors, MyoD and myogenin, which promote commitment and differentiation of cells in the skeletal muscle lineage [18]. Consistent with a cranial mesoderm origin for ESMs, Pax3 was not expressed during development of these muscles and was dispensable for their development in mice [17].

In contrast to Pax3, Pax7 is expressed in skeletal muscle precursors of the esophageal TZ [13, 19–22]. Importantly, however, Pax7 is never expressed in the SMC lineage as shown by lineage tracing studies, immunochemistry, and in situ hybridization [13, 19–22]. Worl et al. used electron microscopy to show that *Pax7*^*−/−*^ mice had a reduced number of skeletal muscle precursor cells in the esophageal ME, defective esophageal skeletal myogenesis, and an aberrantly proximal skeletal-smooth muscle boundary [14]. As *Pax7* is not expressed in the smooth muscle lineage, such mice offer an opportunity to ask whether TZ-based skeletal myogenesis might affect smooth muscle patterning in the esophagus. We report here that *Pax7*^*−/−*^ mice have a severe defect in the postnatal developmental process whereby esophageal smooth muscle is replaced by skeletal muscle. Our results suggest that this defect is due to reduced cell proliferation in the TZ, probably due to precocious differentiation of skeletal muscle precursor cells, leading to a deficit in skeletal muscle. This defect in *Pax7*^*−/−*^ mice is associated with both megaesophagus and defective maturation of esophageal smooth muscle patterning. Our findings add to the previous model for esophageal musculature development. PAX7-dependent cell proliferation in the TZ is important for the proximal-to-distal progression of esophageal skeletal myogenesis. Furthermore, loss of PAX7 leads to a non-autonomous defect that influences smooth muscle fascicle reorientation and development of a mature ME.

## Methods

### Mice

*Pax7*^*+/−*^ mice were originally obtained from Peter Gruss [20] and are from the colony maintained by the Rudnicki laboratory. These mice are on a 129Sv background. It has previously been reported that on a C57BL/6J background, *Pax7*^*+/−*^ esophagi have a mild defect in development of the esophageal musculature [14]. On the 129Sv background, we have not observed differences in esophageal development between *Pax7*^*+/+*^ and *Pax7*^*+/−*^ mice, and at P21 they were indistinguishable in esophageal length, location of the skeletal-smooth muscle boundary, and luminal diameter (Additional file 1: Figure S1). Therefore, we used both *Pax7*^*+/+*^ and *Pax7*^*+/−*^ animals as controls. All animal procedures were conducted in accordance with institutional guidelines for the care and use of laboratory animals as approved by the Institutional Animal Care and Use Committees (IACUC) of the Icahn School of Medicine at Mount Sinai and according to the Canadian Council on Animal Care guidelines and the University of Ottawa Animal Care Committee protocols.

### Preparation of frozen sections

Dissected esophagi were prepared for histology by directly freezing in OCT. For longitudinal sections, incisions were made along the entire esophagus in order to clear out the ingesta, and the sheet of dissected esophagus was laid flat prior to freezing. Frozen tissue blocks were sectioned at 10 μm and placed on Superfrost Plus slides (Thermo Fisher Scientific).

### Immunofluorescence and histology

Frozen sections (10 μm) were immunostained as previously described [13]. Slides were fixed in 4 % PFA, washed in PBS, permeabilized in 0.3 % Triton/PBS, washed in PBS, blocked in 10 % goat serum, and incubated overnight at 4 °C with primary antibodies in blocking buffer. Additional M.O.M. blocking (Vector Laboratories) steps were performed according to the manufacturer’s instructions when mouse primary antibodies were used. Antibodies used included mouse anti-sarcomeric actin (5C5; 1:1000; Sigma-Aldrich), rabbit anti–α-smooth muscle actin (1:200; Abcam), rabbit anti-MyoD (1:500; Santa Cruz Biotechnology, Inc.), mouse anti-myogenin (F5D; 1:50; Santa Cruz Biotechnology, Inc.), mouse anti-M-cadherin (1:75; Santa Cruz Biotechnology, Inc.), rabbit anti-nNOS (C12H1; 1:500; Cell Signaling Technology), rabbit anti-Ki67 (1:1000; Leica), mouse anti-Ki67 (1:100; BD Pharmingen), and rabbit anti-phospho-Histone H3 (ser10) (1:1000; Millipore). After permeabilization, the following antibodies required an antigen retrieval step of boiling the sections in 10 mM sodium citrate: mouse anti-Pax7 (1:100; Developmental Studies Hybridoma Bank, Iowa City, IA), and rabbit cleaved caspase-3 (Asp175; 1:1000; Cell Signaling Technology). Fluorescent secondary antibodies were from Invitrogen and used at 1:1000: goat anti-rabbit Alexa Fluor 488, anti-rabbit Alexa Fluor 568, goat anti-mouse Alexa Fluor 488, and goat anti-mouse IgG1 Alexa Fluor 647. Nuclei were fluorescently labeled with DAPI and mounted with Fluoromount-G anti-fade medium (SouthernBiotech). TUNEL assay was performed using the In Situ Cell Death Detection kit from Roche. For Figs. 4 and 5 and Additional file 2: S2, the transition zone was identified by adjacent longitudinal sections stained with antibodies to 〈-smooth muscle actin and sarcomeric actin.

Hematoxylin and eosin (H&E) staining was performed as described [23]. Sections were dehydrated through graded ethanol and xylene and mounted with Permount (Fisher Scientific).

### Microscopy

Microscopy was performed at the Mount Sinai Microscopy Shared Resource Facility using Axioplan2 and Axioplan2 IE microscopes (Carl Zeiss) equipped with ×10, ×20, ×40, and ×100 objective lenses that had numerical apertures of 0.5, 0.8, 0.75, and 1.4, respectively. Images were acquired at room temperature with a camera (Axio Cam MRm; Carl Zeiss). Mosaic images were compiled using the stitching feature in the AxioVision software. The maximum luminal diameter and length of esophageal skeletal/smooth muscle were measured by using ImageJ software (National Institutes of Health).

### Statistical analysis

Data were expressed as mean ± standard deviation. Differences were tested by two-tailed *t* test. Values with *P* < 0.05 were considered statistically significant.

## Results

### *Pax7*^*−/−*^ mice develop megaesophagus

**Fig. 1:**
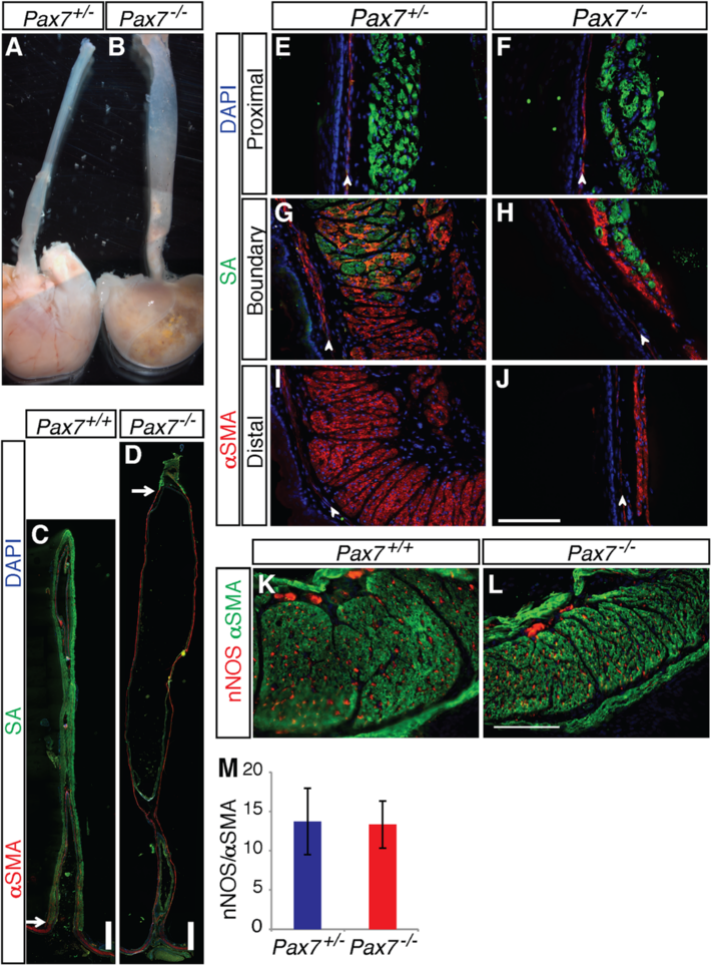
*Pax7* ^*−/−*^ mice have megaesophagus. **a**, **b** P21 *Pax7* ^*+/−*^ and *Pax7* ^*−/−*^ esophagi. **c**–**j** Longitudinal sections of P21 *Pax7* ^*+/−*^ and *Pax7* ^*−/−*^ esophagi were stained with antibodies to α-smooth muscle actin (αSMA; *red*) and sarcomeric actin (SA; *green*) for smooth and skeletal muscles, respectively. **c**, **d** *Pax7* ^*−/−*^ esophagi have an aberrantly proximally located skeletal-smooth muscle boundary (*arrows*). Note that the *Pax7* ^*+/+*^ esophagus is cut off at the top end, making it appear shorter than the *Pax7* ^*−/−*^ esophagus, the full length of which is shown. In fact, at P21, *Pax7* ^*−/−*^ esophagi are shorter than *Pax7* ^*+/+*^ esophagi (see Fig. 3e). **e**, **f** The proximal ME of *Pax7* ^*−/−*^ esophagi has skeletal muscle of relatively normal appearance. **g**, **h** Smooth muscle cells are dispersed with skeletal myofibers around the skeletal-smooth muscle boundary (*Boundary*). Note that the ME of the *Pax7* ^*−/−*^ esophagus is thinner than the control at the skeletal-smooth muscle boundary. **i**, **j** The distal *Pax7* ^*−/−*^ esophagus has a long, thin extension of smooth muscle instead of the short, broad segment of smooth muscle found in controls. **e**–**j** The thin αSMA^*+*^ layer adjacent to the ME is the muscularis mucosa (*arrowheads*). **k**, **l** Longitudinal sections through the LES of P21 *Pax7* ^*+/+*^ and *Pax7* ^*−/−*^ esophagi mice were stained with antibodies to αSMA (*green*) and nNOS (*red*) to label inhibitory intramural neurons. **m** Quantification of the relative numbers of nNOS^*+*^ cells to αSMA staining in the ME of the LES. Values are means ± SD, *n* = 5. *Bars*: (**c**, **d**) 1 mm; (**e**–**l**) 0.2 mm

Most *Pax7*^*−/−*^ mice die within 3 weeks of birth [20]. Surviving P21 *Pax7*^*−/−*^ mice displayed megaesophagus with 100 % penetrance. P21 *Pax7*^*−/−*^ esophagi had an enlarged diameter relative to *Pax7*^*+/+*^ and *Pax7*^*+/−*^ esophagi, and a dilated lumen filled with partially digested food (Fig. 1a–d). No other gross abnormalities were observed in the GI tracts of *Pax7*^*−/−*^ mice. P21 *Pax7*^*+/−*^ esophagi resembled the wild type in esophageal length, location of the skeletal-smooth muscle boundary, and luminal diameter (Additional file 1: Figure S1); therefore, *Pax7*^*+/+*^ and *Pax7*^*+/−*^ esophagi were used interchangeably as controls. Immunofluorescence analysis (IFA) of longitudinal sections of P21 esophagi with markers of smooth muscle (α-smooth muscle actin (αSMA)) and skeletal muscle (sarcomeric actin (SA)) revealed that *Pax7*^*−/−*^ mice have a mispatterned ME, with the skeletal-smooth muscle boundary occurring at an abnormally proximal position (Fig. 1c, d). Most smooth muscle in the esophagi of *Pax7*^*+/−*^ mice was found in a short, broad segment at the esophagogastric junction, where SMCs were bundled into fascicles that were stacked side by side with an orientation perpendicular to the lumen (Fig. 1i). In contrast, distal to the aberrantly proximal boundary in *Pax7*^*−/−*^ esophagi, there was a long, thin, ectopic extension of smooth muscle that connected to the LES (Fig. 1d, j). The fascicles in this ectopic region of smooth muscle in *Pax7*^*−/−*^ esophagi were parallel to the lumen, indicating they had failed to undergo the normal reorientation process. A few SMCs were dispersed within the skeletal muscle at the skeletal-smooth muscle boundary in both *Pax7*^*+/−*^ and *Pax7*^*−/−*^ esophagi (Fig. 1g, h). Despite the mispatterned and thinner ME distal to the aberrantly proximal boundary in *Pax7*^*−/−*^ esophagi, the morphology of the smooth muscle fascicles appeared normal in the *Pax7*^*−/−*^ LES (Fig. 1k, l). The *Pax7*^*−/−*^ LES was somewhat thinner than that of the control mice (control = 135.8 ± 9.6 mm (*n* = 5); mutant = 92.0 ± 12.9 mm (*n* = 6); *p* < 0.01). This may be due to a combination of defective development of the ME and the overall growth retardation displayed by these mice [20]. The ME of the proximal portion of *Pax7*^*−/−*^ esophagi was similar to that of *Pax7*^*+/−*^ esophagi, although the skeletal myofibers in mutants appeared to have a greater amount of between-fiber space than in controls (Fig. 1f).

Megaesophagus is sometimes associated with loss of nitrergic neurons in the myenteric plexus of the LES [3]. We therefore quantified the number of neurons in the LES from P21 *Pax7*^*+/+*^ and *Pax7*^*−/−*^ mice. IFA of LES sections with an antibody to nNOS revealed that the number and pattern of nitrergic neurons were similar between control and mutant mice (Fig. 1k–m). Taken together, P21 *Pax7*^*−/−*^ mice showed megaesophagus with an aberrantly proximal skeletal-smooth muscle boundary. The morphological defects of the P21 *Pax7*^*−/−*^ ME were most apparent in the region between the skeletal-smooth muscle boundary and LES, but were not as severe in the proximal region and in the LES itself.

### Defective ME patterning in *Pax7*^*−/−*^ esophagi

**Fig. 2:**
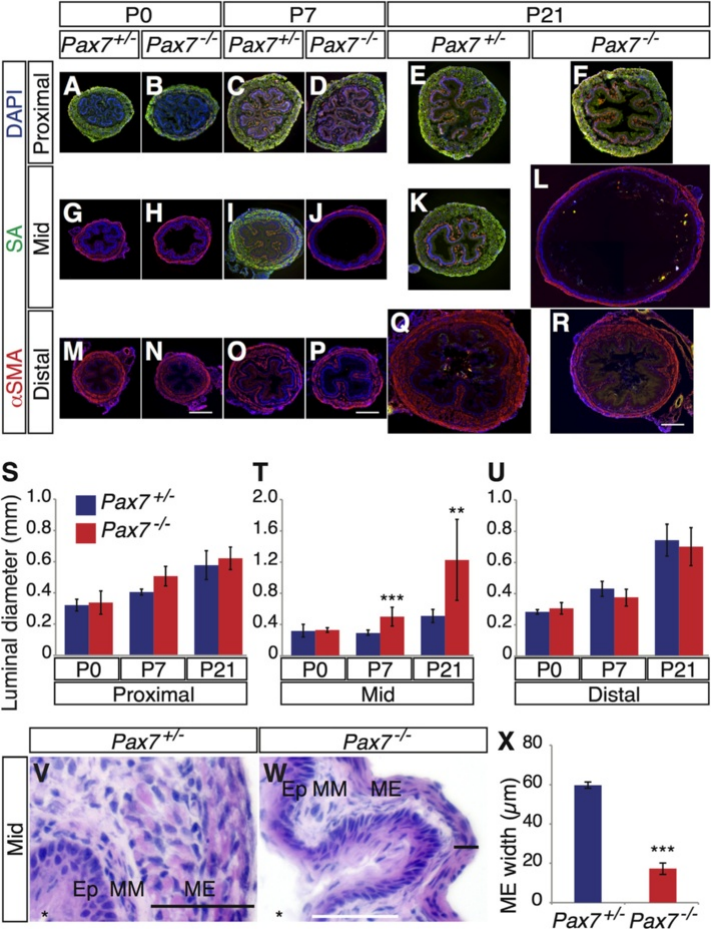
Defects in ME patterning in *Pax7* ^*−/−*^ esophagi. **a**–**r** Cross sections of P0, P7, and P21 *Pax7* ^*+/−*^ and *Pax7* ^*−/−*^ esophagi were stained with antibodies to αSMA, SA, and DAPI. **j**, **l** The P7 and P21 *Pax7* ^*−/−*^ esophagi are dilated, and skeletal muscle is replaced by smooth muscle at the mid-level region (Mid). **s**–**u** Luminal diameter at the mid region of *Pax7* ^*−/−*^ esophagi is larger than that of *Pax7* ^*+/−*^ esophagi at P7 and P21. Values are means ± SD, *n* = 3–11. **v**, **w** Cross sections of P7 *Pax7* ^*+/−*^ and *Pax7* ^*−/−*^ esophagi at the mid region were stained with H&E. *Ep* epithelium, *MM* muscularis mucosa, *ME* muscularis externa. The *black horizontal bars* indicate the width of the ME; the *asterisks* indicate the lumens. **x** Quantification of the width of the ME, as shown in (**v** and **w**). Values are means ± SD, *n* = 3. *Bars*: (**a**–**r**) 0.2 mm, (**v**, **w**) 50 μm. **P < 0.01; ***P < 0.001

To determine when these defects in *Pax7*^*−/−*^ mice arose, cross sections were taken from P0-P21 at proximal, mid-level, and distal regions. Expression of αSMA and SA was assessed. The proximal and distal ME were composed of skeletal and smooth muscles, respectively, and the cross sections of proximal and distal *Pax7*^*−/−*^ esophagi showed no obvious defects at P0, P7, and P21 (Fig. 2a–f, m–r, s, u). Mid-level cross sections revealed that esophageal dilation became recognizable in *Pax7*^*−/−*^ esophagi at P7 (Fig. 2g–j, t), and this distention was more severe at P21 (Fig. 2k, l, t). H&E-stained cross sections revealed that the P21 Pax7^−/−^ ME was thinner than that of controls in the mid-level region (Fig. 2v–x). In contrast to the control mid-level ME, which was composed of skeletal muscle at P7 and P21, the *Pax7*^*−/−*^ mid-level ME displayed only smooth muscle at these time points (Fig. 2i–l).

**Fig. 3:**
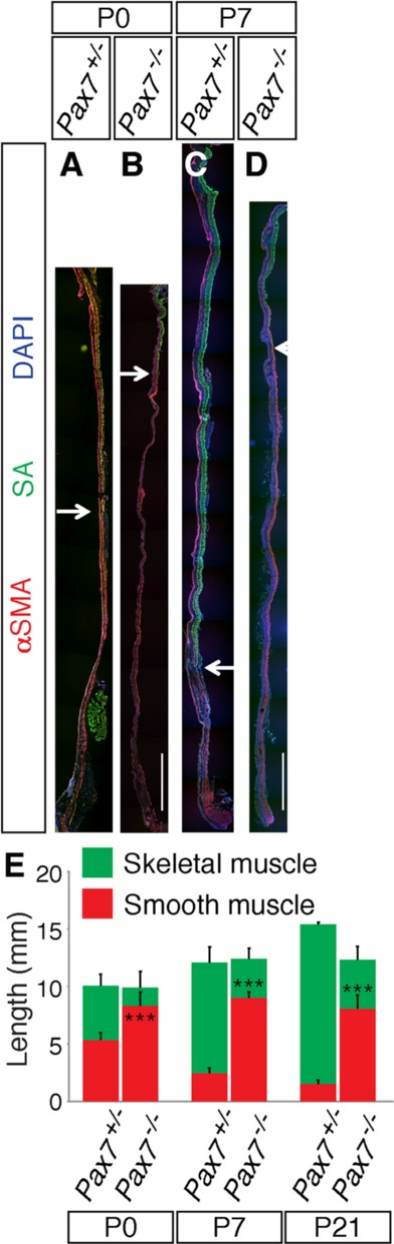
*Pax7* ^*−/−*^ mice display defective proximal-to-distal progression of ME development. **a**–**d** Longitudinal sections of P0 and P7 *Pax7* ^*+/−*^ and *Pax7* ^*−/−*^ esophagi were stained with antibodies to αSMA, SA, and DAPI. *Pax7* ^*−/−*^ esophagi have an aberrantly proximally located skeletal-smooth muscle boundary (*arrows*). The SA^+^ tissue near the distal end of the *Pax7* ^*+/−*^ esophagus in (**a**) is the diaphragm. **e** The distance between the distal-most SA^*+*^ cell and the LES was measured and is represented by the *red portion* of the histogram bars. The distance decreases progressively with age in *Pax7* ^*+/−*^ esophagi, but this fails to occur in *Pax7* ^*−/−*^ esophagi. Values are means ± SD, *n* = 3–5. *Bars*: 1 mm. ***P < 0.001

These findings are consistent with the aberrant skeletal-smooth muscle boundary seen in P21 *Pax7*^*−/−*^ esophagi (Fig. 1d), and they suggested a defect in the process whereby smooth muscle is replaced by skeletal muscle in these animals. We therefore analyzed the proximal-to-distal progression of skeletal myogenesis during ME development. Expression of αSMA and SA was assessed in longitudinal sections taken at P0, P7, and P21 (Figs. 1c, d and 3a–d). The distal-most skeletal muscle cells in these sections were embedded in smooth muscle. Progression of ME development was monitored by measuring the distance from the distal-most SA^+^ cell to the LES. At P0, this distance was about half of the length in *Pax7*^*+/−*^ esophagi, whereas it constituted nearly 80 % of the total length in *Pax7*^*−/−*^ esophagi (Fig. 3a, b, e). The distance from the distal-most SA^+^ cell to the LES diminished in *Pax7*^*+/−*^ esophagi from P0 to P21, and the distal-most SA^+^ cell was found just proximal to the LES at P21 (Figs. 1c and 3a, c, e). In contrast, the distal-most SA^+^ cell in *Pax7*^*−/−*^ animals was at a significantly more proximal location than in *Pax7*^*+/−*^ animals as early as P0 and maintained through P21 (Figs. 1d and 3b, d, e). The distance from the distal-most SA^+^ cell to the LES slightly decreased in *Pax7*^*−/−*^ esophagi by P21; however, the distance still constituted ~65 % of the total length (Fig. 3e). The total length of *Pax7*^*−/−*^ esophagi is ~20 % shorter than that of control animals by P21 (Fig. 3e) [14]. This is likely due to overall growth retardation in these mice [20], but cannot alone account for the ME patterning defects.

### *Pax7*^*−/−*^ esophagi have reduced number of cells undergoing myogenic differentiation

**Fig. 4:**
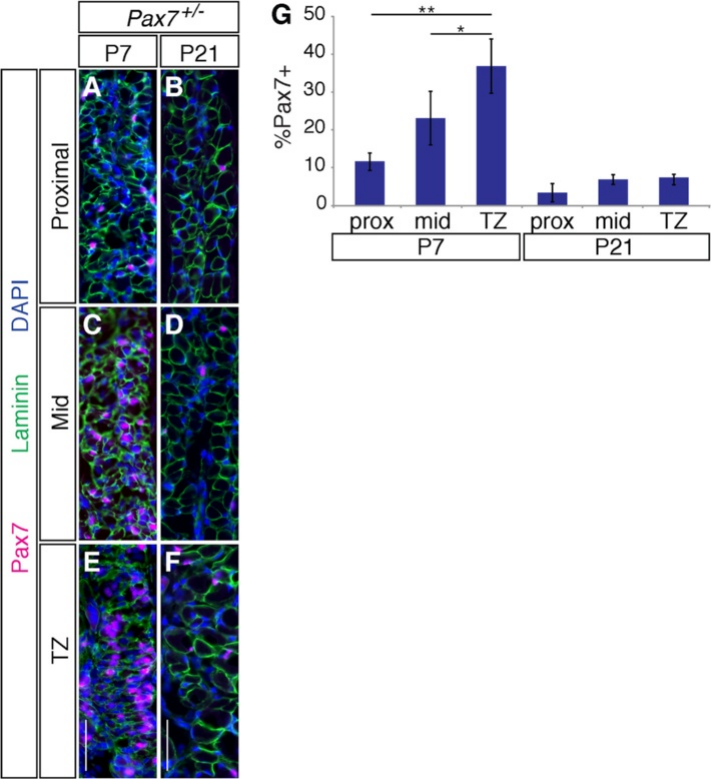
Expression of Pax7 in the esophageal musculature at P7 and P21. **a**–**f** Longitudinal sections of P7 and P21 *Pax7* ^*+/−*^ esophagi were stained with antibodies to Pax7, laminin, and DAPI. Proximal, mid-level (*Mid*), and TZ regions were analyzed. The greatest number of Pax7^+^ cells was observed in the TZ at P7, and the numbers diminished in mid and proximal regions. The number of Pax7^+^ cells is diminished at P21 when proximal-to-distal progression of ME development is complete. **g** Quantification of Pax7^+^ cells in proximal (*prox*), mid, and TZ regions measured as a percentage of total DAPI^+^ cells in the ME. *Bars*: 0.2 mm

To analyze the Pax7 expression pattern in developing esophageal ME, longitudinal sections of *Pax7*^*+/−*^ esophagi were taken at P7 and P21 and stained with an antibody for Pax7. At P7, most Pax7^+^ cells were found in the TZ, and their number decreased at more proximal locations (Fig. 4a, c, e, g). Pax7^+^ cells diminished from the entire esophagi at P21, when proximal-to-distal progression of skeletal muscle was complete (Fig. 4b, d, f, g). At this stage, relatively few Pax7^+^ cells were present, as these probably mainly represent the population of satellite cells seen in the adult esophagus [13, 24].

**Fig. 5:**
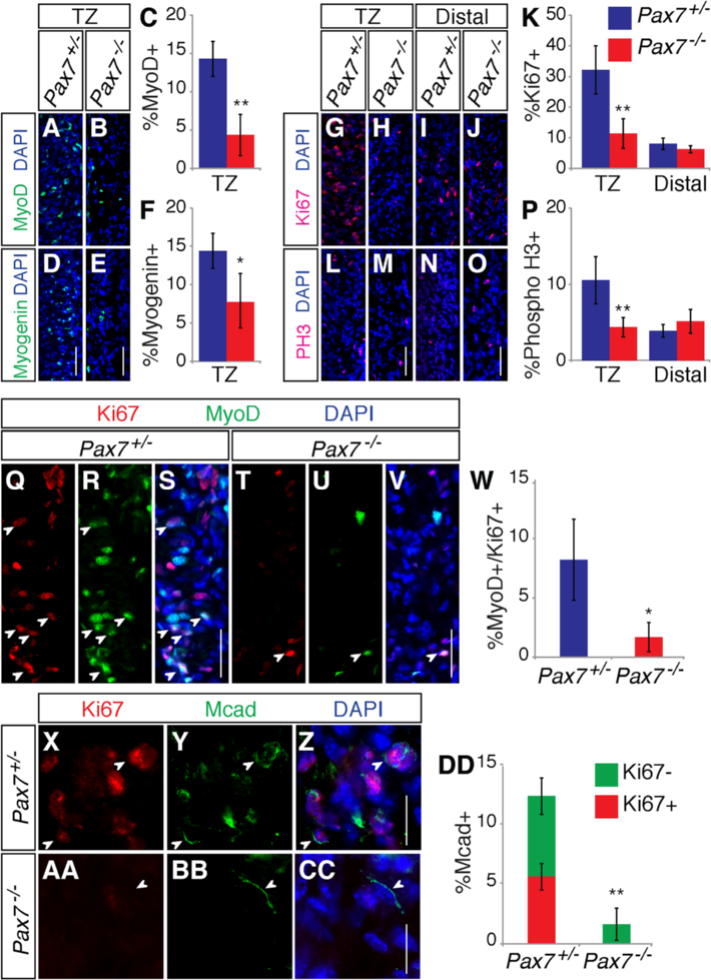
Reduced skeletal muscle precursor cell proliferation in the *Pax7* ^*−/−*^ TZ. **a**, **b**, **d**, **e** Longitudinal sections of P7 *Pax7* ^*+/−*^ and *Pax7* ^*−/−*^ esophagi were stained with either MyoD (**a**, **b**) or Myogenin (**d**, **e**) antibodies and DAPI. The TZ was analyzed. **c, f** Quantification of MyoD^+^ (**c**) and Myogenin^+^ (**d**) cells within the TZ, measured as a percentage of total DAPI^+^ cells in the ME. Values are means ± SD, *n* = 3–5. **g**–**j**, **l**–**o** Longitudinal sections of P7 *Pax7* ^*+/−*^ and *Pax7* ^*−/−*^ esophagi were stained with either Ki67 (**g**–**j**) or phospho-histone H3 (PH3) (**l**–**o**) and DAPI. The TZ and distal region were analyzed. **k**, **p** Quantification of Ki67^+^ (**k**) and PH3^+^ (**p**) cells within the TZ and distal region, measured as a percentage of total DAPI^+^ cells in the given region. The numbers of Ki67^+^ and PH3^+^ cells were reduced in the TZ in *Pax7* ^*−/−*^ mice, but not in the distal region. Values are means ± SD, *n* = 4–5. **q**–**v** Longitudinal sections of P7 *Pax7* ^*+/−*^ and *Pax7* ^*−/−*^ esophagi were stained with MyoD and Ki67 antibodies and with DAPI. **w** Quantification of MyoD^+^/Ki67^+^ cells within the TZ, measured as a percentage of total DAPI^+^ cells in the TZ. Values are means ± SD, *n* = 4. **x**–**cc** Longitudinal sections of P7 *Pax7* ^*+/−*^ and *Pax7* ^*−/−*^ esophagi were stained with M-cadherin (Mcad) and Ki67 antibodies and with DAPI. **dd** Quantification of total Mcad^+^ and Mcad^+^/Ki67^+^ cells within the TZ, measured as a percentage of total DAPI^+^ cells in the TZ. Values are means ± SD, *n* = 4. *Bars*: 0.2 mm (**a**–**v**); 20 μm (**x**–**cc**). *P < 0.05; **P < 0.01

We hypothesized that impaired proximal-to-distal progression of skeletal myogenesis in *Pax7*^*−/−*^ esophagi resulted from a decrease in the number of cells undergoing myogenic differentiation. We therefore assessed the expression of MyoD (a marker of determined myoblasts) and myogenin (a marker of differentiating myoblasts) during ME development. Longitudinal sections of P7 *Pax7*^*+/−*^ and *Pax7*^*−/−*^ esophagi were stained with antibodies for MyoD and myogenin. Consistent with our previous study [13], most MyoD^+^ and myogenin^+^ cells were found in the TZ and their numbers diminished at more proximal locations in control animals. *Pax7*^*−/−*^ mice had ~30 % the number of MyoD^+^ cells and ~50 % the number of myogenin^+^ cells as control mice (Fig. 5a–f).

Reduction of the number of cells expressing these markers of myogenesis may result from a decrease in the muscle progenitor cell population, either by alteration in cell proliferation or cell survival. To investigate cell proliferation in the developing ME, longitudinal sections were taken from P7 *Pax7*^*+/−*^ and *Pax7*^*−/−*^ esophagi and stained with antibodies for Ki67 and phospho-histone H3. Most Ki67^+^ and phospho-histone H3^+^ cells were found in the TZ in *Pax7*^*+/−*^ esophagi (Fig. 5g, k, l, p). Much lower numbers of Ki67^+^ and phospho-histone H3^+^ cells were found in the *Pax7*^*−/−*^ TZ (Fig. 5h, k, m, p). Consistent with the largely postmitotic nature of ME smooth muscle cells [13], fewer Ki67^+^ and phospho-histone H3^+^ cells were present in the distal ME below the TZ in both *Pax7*^*+/−*^ and *Pax7*^*−/−*^ esophagi (Fig. 5i, j, k, n, o, p). Therefore, cell proliferation in the TZ is substantially reduced in *Pax7*^*−/−*^ mice.

To confirm that cell proliferation was reduced in skeletal muscle precursor cells per se, we stained longitudinal P7 sections with antibodies for MyoD or another lineage marker, M-cadherin, plus Ki67. *Pax7*^*−/−*^ mice had ~20 % the number of MyoD^+^/Ki67^+^ cells in the TZ as control mice (Fig. 5q–w). *Pax7*^*−/−*^ mice had only ~13 % the number of M-cadherin^+^ cells as controls (Fig. 5x–dd). Furthermore, ~45 % of control M-cadherin^+^ cells were also positive for Ki67, whereas none of the small number of M-cadherin^+^ cells in the *Pax7*^*−/−*^ TZ were also positive for Ki67. These results indicate that cell proliferation of skeletal muscle precursors is strongly diminished in the *Pax7*^*−/−*^ esophagus.

We next analyzed longitudinal sections of P7 *Pax7*^*+/−*^ and *Pax7*^*−/−*^ esophagi by TUNEL assay and by IFA with an antibody to cleaved caspase-3, with thymuses as positive controls. TUNEL^+^ and cleaved caspase-3^+^ cells were easily detected in control thymuses (Additional file 2: Figure S2E, J). Consistent with previous studies [7–9, 13], no apoptotic cells were observed in *Pax7*^*+/−*^ esophagi (Additional file 2: Figure S2A, C, F, H). Furthermore, no TUNEL^+^ or cleaved caspase-3^+^ cells were observed in the esophagi of *Pax7*^*−/−*^ mice (Additional file 2: Figure S2B, D, G, I), indicating that the diminished skeletal myogenesis seen in these mice is not a consequence of cell death.

## Discussion

In this study, we investigated the role of *Pax7* in esophageal musculature development. *Pax7*^*−/−*^ mice displayed megaesophagus with a severe defect in the postnatal developmental processes whereby esophageal smooth muscle is replaced by skeletal muscle and the ME acquires a mature pattern. The proximal-to-distal progression of skeletal myogenesis was impaired in *Pax7*^*−/−*^ esophagi, associated with a significant reduction in cell proliferation of skeletal muscle precursors in the TZ. Additionally, our findings revealed that Pax7 is important for proper postnatal patterning of the entire ME, including the distal smooth muscle layers.

We previously proposed a model for postnatal development of the esophageal ME [13]. During esophageal ME maturation, skeletal muscle progenitors commit to the myogenic lineage and differentiate within a TZ that migrates along the length of the esophagus in a proximal-to-distal manner. Distal to the TZ, SMCs are largely non-proliferative and bundled into long, thin fascicles. SMC fascicles are initially arranged end-to-end and parallel to the lumen. During ME maturation, SMC fascicles rearrange their orientation such that they are ultimately arranged side by side and nearly perpendicular to the lumen; as a consequence, at the end of ME maturation, the fascicles occupy only the distal portion of the ME. Fascicular reorientation occurs in a distal-to-proximal manner. We suggested that this process “clears a path” for the TZ, which migrates distally and produces the skeletal myofibers that constitute the majority of the length of the mature ME of the adult mouse [13].

Mice lacking the multifunctional cell surface receptor, Cdo, have a mispatterned ME, including an aberrantly proximal skeletal-smooth muscle boundary, and they develop megaesophagus. These mice are specifically defective in SMC fascicular reorientation, without obvious alterations in TZ-based skeletal myogenesis [13]. *Cdo*^*−/−*^ mice had an ectopic, proximal region of smooth muscle in which the fascicles remained in the end-to-end, parallel-to-the-lumen orientation that normally characterizes an earlier developmental stage. This phenotype is very likely to be SMC-autonomous [13]. In contrast, the esophageal defects in *Pax7*^*−/−*^ mice are almost certainly autonomous to the skeletal muscle lineage. *Pax7* is expressed in skeletal muscle precursor cells throughout the body, including the esophageal TZ, but it is never expressed in the SMC lineage [13, 19–22]. Examination of esophageal ME patterning in *Pax7*^*−/−*^ esophagi revealed that their musculature consisted mainly of smooth muscle, having skeletal muscle only at the very proximal end. This phenotype was more severe in *Pax7*^*−/−*^ esophagi than *Cdo*^*−/−*^ esophagi. Smooth muscle occupied the distal ~20 % of P14 *Cdo*^*−/−*^ esophagi, whereas it occupied the distal ~65 % of P21 *Pax7*^*−/−*^ esophagi. There was no difference in the position of the skeletal-smooth muscle boundary between control and *Cdo*^*−/−*^ mice at P0, but *Pax7*^*−/−*^ esophagi already had an aberrantly proximal skeletal-smooth muscle boundary at P0. Moreover, in contrast to *Cdo*^*−/−*^ mice, the numbers of MyoD^+^ and myogenin^+^ cells were reduced, as was cell proliferation, in the *Pax7*^*−/−*^ TZ. Therefore, the aberrantly proximal skeletal-smooth muscle boundary in *Pax7*^*−/−*^ esophagi resulted from reduced production of ESM.

Distal to the aberrantly proximal skeletal-smooth muscle boundary, the *Pax7*^*−/−*^ smooth muscle layer was very thin, with the fascicles parallel to the lumen for nearly the entire length of the ME. The LES was normally patterned, consistent with it being in the region in which fascicles reorient earliest [13]. As *Pax7* is never expressed in the SMC lineage, the mispatterning of ESM in *Pax7*^*−/−*^ esophagi is presumably a non-cell-autonomous effect. It seems likely that smooth muscle mispatterning occurs as a consequence of impaired proximal-to-distal progression of the *Pax7*^*−/−*^ TZ, but further work will be required to prove this. One physiological consequence of this combination of deficient skeletal myogenesis and SMC fascicle mispatterning is that *Pax7*^*−/−*^ mice have an enlarged luminal diameter specifically in the mid region of the esophagus (i.e., megaesophagus). We speculate that smooth muscles, in this abnormal location and fascicular arrangement, are not successful in maintaining the full integrity and function of the ME. This may occur for a variety of non-mutually exclusive reasons, including defective peristalsis, causing the slow passage of food and physical distension; suboptimal innervation; or some combination of these and additional processes.

Our findings support and add to the previous model for esophageal musculature development. Based on the fact that *Pax7*^*−/−*^ mice display a presumably non-SMC-autonomous defect in fascicular reorientation, we propose the following hypothesis. Smooth muscle fascicular reorientation may be facilitated by forces exerted via the proximal-to-distal progression of the TZ. This progression may “push” the SMCs to a more distal region, where they are eventually exposed to the signals that trigger the rearrangement of SMCs relative to one another, resulting in reorientation of fascicles. Proliferation of skeletal muscle precursor cells was significantly reduced in the *Pax7*^*−/−*^ TZ during esophageal muscular development. This suggests that robust cell proliferation, which presumably participates in driving the proximal-to-distal progression of the TZ, depends on PAX7. This is consistent with the known roles of PAX7 in maintaining proliferation and preventing precocious differentiation [24, 25]. Therefore, it is most likely that skeletal muscle precursor cells differentiate prematurely during esophageal myogenesis in the Pax7^−/−^ TZ, resulting in reduced production of ESM.

That robust cell proliferation in the TZ may be critical for the proximal-to-distal progression of skeletal myogenesis is supported by a mathematical model of cell migration [26]. This model predicts that proliferation at the invading front is the key mechanism driving directed migration. These predictions were experimentally validated with the directional migration and colonization of the gut by vagal neural crest cells that establish the enteric nervous system [26]. This “frontal expansion” model, which emphasizes the importance of proliferation to a carrying capacity, is potentially relevant to a wider range of invasion systems such as angiogenesis, epidermal wound healing, and malignant invasion [26]. Therefore, this model may also apply to the proximal-to-distal progression of esophageal skeletal myogenesis.

Megaesophagus is observed as a feature of several animal models of Duchenne muscular dystrophy, including some strains of *mdx* mice, and some dystrophic dog and cat models [27–30]. It was proposed that megaesophagus in some of these animal models was caused by a hypertrophic diaphragm and ensuing esophageal constriction, a very different scenario than that seen in *Pax7*^*−/−*^ mice. There is, however, little in the literature to suggest that megaesophagus is common in patients with muscular dystrophy or other myopathies. Megaesophagus occurs more frequently in severe cases of achalasia, as a consequence of inability to fully relax the LES [2, 3]. It will be of interest for future studies to use electrophysiology and manometry to test if *Pax7*^*−/−*^ mice have a non-autonomous defect in LES function.

## Conclusions

Our analyses on *Pax7*^*−/−*^ esophageal musculature development suggest that ME patterning requires robust, PAX7-dependent cell proliferation in the TZ, which helps drive the proximal-to-distal progression of skeletal myogenesis. This process may in turn be necessary for distal smooth muscle fascicle reorientation and development of a mature ME.

## Additional files

Additional file 1: Figure S1.
*Pax7* ^*+/+*^ and *Pax7* ^*+/−*^ esophagi have similar luminal diameter, esophageal length, and location of the skeletal-smooth muscle boundary. **(A)** Cross-sections of mid-region (Mid) esophagi from P21 mice of the indicated genotype were used to quantify luminal diameters. Values are means ± SD, n = 3. **(B)** Longitudinal sections of P21 *Pax7* ^*+/+*^ and *Pax7* ^*+/−*^ esophagi were stained with antibodies to αSMA and SA and with DAPI. The length of the entire esophagus was measured, as was the location of the skeletal-smooth muscle boundary. Values are means ± SD, n = 3. *Pax7* ^*+/+*^ and *Pax7* ^*+/−*^ esophagi were not different in these parameters. Additional file 2: Figure S2. Lack of apoptosis in control or *Pax7* ^*−/−*^ esophageal musculature at P7. **(A-D and F-I)** Longitudinal sections of P7 *Pax7* ^*+/−*^ and *Pax7* ^*−/−*^ esophagi were stained with antibodies to cleaved caspase 3 (C-caspase 3) (A-D) and DAPI, or for TUNEL^+^ cells (F-I) and DAPI. The TZ and distal region were analyzed. Apoptotic cells were not observed in the esophageal ME. **(E and J)** *Pax7* ^*+/−*^ thymuses were used as a positive control and displayed easily detectable apoptotic cells by both IFA for cleaved caspase 3 (E) and TUNEL (J). Bars: 0.2 mm.

## Abbreviations

CDO: cell adhesion molecule-related/down-regulated by oncogenes
ESM: esophageal skeletal muscle
GERD: gastroesophageal reflux disease
IFA: immunofluorescence analysis
LES: lower esophageal sphincter
ME: muscularis externa
Myf5: myogenic factor 5
MyoD: myoblast determination protein
nNOS: neuronal nitric oxide synthases
NO: nitric oxide
PAX: paired box protein
SA: sarcomeric actin
SMA: smooth muscle actin
SMC: smooth muscle cell
TZ: transition zone
