# Supplementary figures and images for: PAX7 is required for patterning the esophageal musculature
Source: Skelet Muscle. 2015 Dec 3;5:39. doi: 10.1186/s13395-015-0068-0 (PMC4668666; doi:10.1186/s13395-015-0068-0)

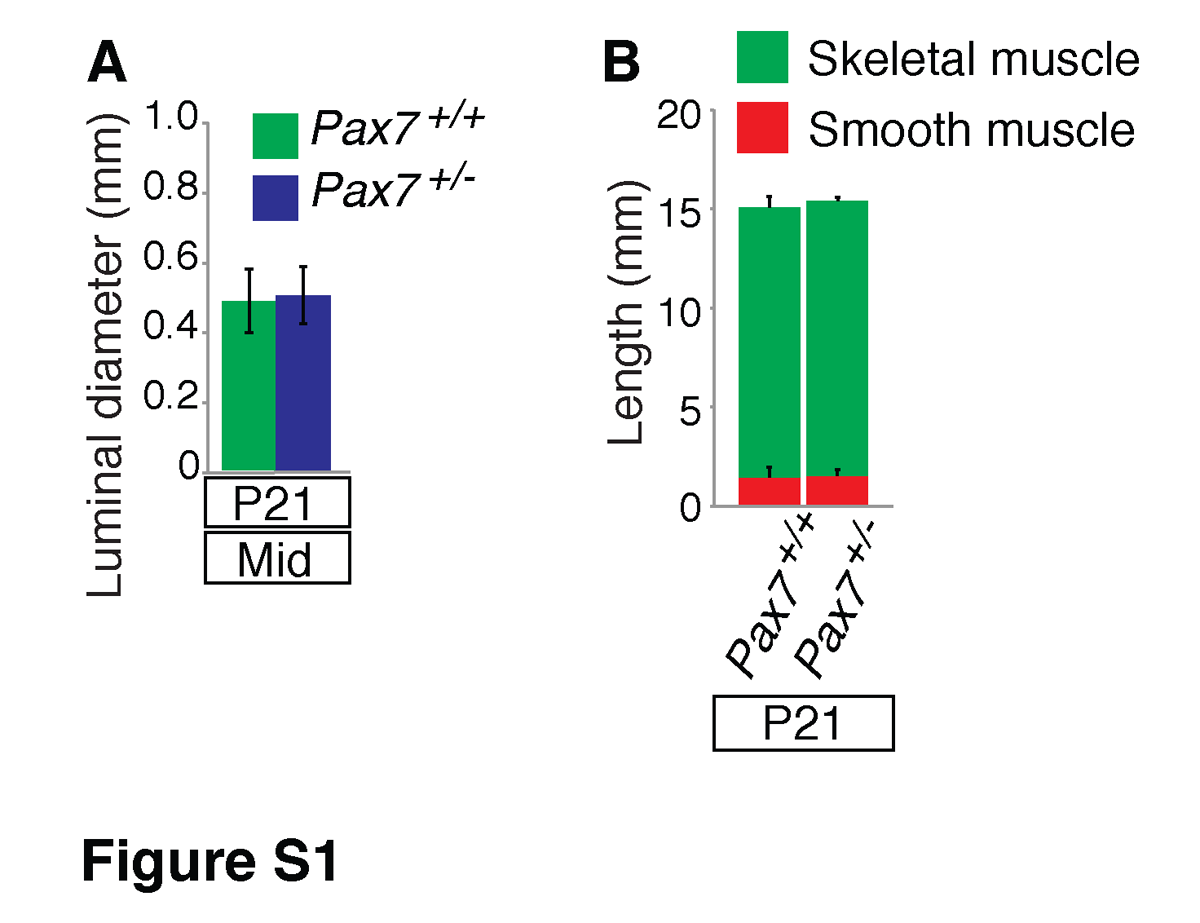

Supplement: Additional file 1: Figure S1. — Pax7 +/+ and Pax7 +/− esophagi have similar luminal diameter, esophageal length, and location of the skeletal-smooth muscle boundary. (A) Cross-sections of mid-region (Mid) esophagi from P21 mice of the indicated genotype were used to quantify luminal diameters. Values are means ± SD, n = 3. (B) Longitudinal sections of P21 Pax7 +/+ and Pax7 +/− esophagi were stained with antibodies to αSMA and SA and with DAPI. The length of the entire esophagus was measured, as was the location of the skeletal-smooth muscle boundary. Values are means ± SD, n = 3. Pax7 +/+ and Pax7 +/− esophagi were not different in these parameters. [file 13395_2015_68_MOESM1_ESM.tif]

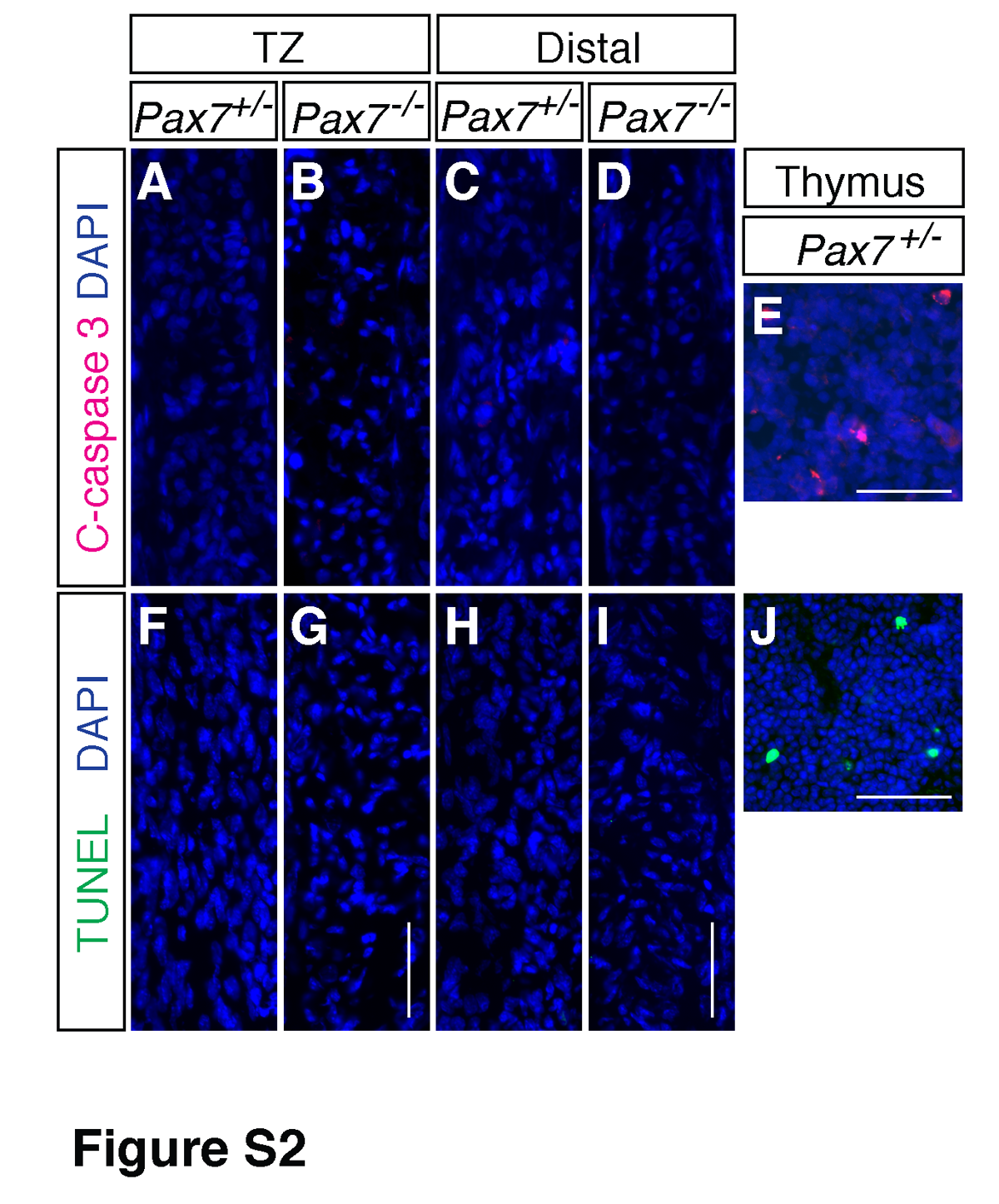

Supplement: Additional file 2: Figure S2. — Lack of apoptosis in control or Pax7 −/− esophageal musculature at P7. (A-D and F-I) Longitudinal sections of P7 Pax7 +/− and Pax7 −/− esophagi were stained with antibodies to cleaved caspase 3 (C-caspase 3) (A-D) and DAPI, or for TUNEL+ cells (F-I) and DAPI. The TZ and distal region were analyzed. Apoptotic cells were not observed in the esophageal ME. (E and J) Pax7 +/− thymuses were used as a positive control and displayed easily detectable apoptotic cells by both IFA for cleaved caspase 3 (E) and TUNEL (J). Bars: 0.2 mm. [file 13395_2015_68_MOESM2_ESM.tif]
